# Editorial: Detection and Estimation of Working Memory States and Cognitive Functions Based on Neurophysiological Measures

**DOI:** 10.3389/fnhum.2018.00440

**Published:** 2018-10-30

**Authors:** Felix Putze, Christian Mühl, Fabien Lotte, Stephen Fairclough, Christian Herff

**Affiliations:** ^1^Cognitive Systems Lab, University of Bremen, Bremen, Germany; ^2^Sleep and Human Factors Research, German Aerospace Center, Institute of Aerospace Medicine, Cologne, Germany; ^3^Inria / LaBRI (CNRS - Bordeaux INP - Univ. Bordeaux), Talence, France; ^4^School of Natural Sciences and Psychology, Liverpool John Moores University, Liverpool, United Kingdom; ^5^School for Mental Health and Neuroscience, Maastricht University, Maastricht, Netherlands

**Keywords:** cognitive functions, working memory - long-term memory interactions, BCI (brain computer interface), EEG, fNIRS (functional near infrared spectroscopy), cognitive processes

## 1. Scope

Executive cognitive functions like working memory determine the success or failure of a wide variety of different cognitive tasks, such as problem solving, navigation, or planning. Estimation of constructs like working memory load or memory capacity from neurophysiological or psychophysiological signals would enable adaptive systems to respond to cognitive states experienced by an operator and trigger responses designed to support task performance (e.g., by simplifying the exercises of a tutor system when the subject is overloaded Gerjets et al., 2014, or by shutting down distractions from the mobile phone). The determination of cognitive states like working memory load is also useful for automated testing/assessment or for usability evaluation. While there exists a large body of research work on neural and physiological correlates of cognitive functions like working memory activity, fewer publications deal with the application of this research with respect to single-trial detection and real-time estimation of cognitive functions in complex, realistic scenarios. Single-trial classifiers based on brain activity measurements such as electroencephalography (EEG, Kothe and Makeig, 2011; Lotte et al., 2018), functional near-infrared spectroscopy (fNIRS, Putze et al., 2014; Herff et al., 2015), physiological signals (Fairclough et al., 2005; Fairclough, 2008), or eye tracking (Putze et al., 2013) have the potential to classify affective (Koelstra et al., 2010; Heger et al., 2014; Mühl et al., 2014) or cognitive states based upon short segments of data. For this purpose, signal processing and machine learning techniques need to be developed and transferred to real-world user interfaces.

The goal of this Frontiers Research Topic was to advance the State-of-the-Art in signal-based modeling of cognitive processes. We were especially interested in research toward more complex and realistic study designs, for example collecting data in the wild or investigating the interaction between different cognitive processes or signal modalities. Bringing together many contributions in one format allowed us to look at the state of convergence or diversity regarding concepts, methods, and paradigms.

The accepted manuscripts in this research topic cover a large range of aspects of cognition, reflecting the broadness of the field and its many application domains. A dominant challenge in the research topic is the analysis of cognitive workload (or memory load) from neurological signals. This does not come as a surprise because workload is a thoroughly studied construct and workload models can be immediately exploited, e.g., for adaptive human-machine interaction. While all these manuscripts share a joint research interest, they tackle the challenge of workload modeling in different application domains, with different signals, different classification approaches, and different features. Working memory and attentional control represent two recurring themes through the collection of papers in this research theme. The most prominent modality in this research topic is EEG, drawing from both spectral as well as time-domain features. In multiple articles, EEG is complemented by other modalities: Two of them use fNIRS as a different mode to capture neural activity (two others use fNIRS as single modality); two others use eye tracking as a way to capture visual attention and one also uses physiological signals (such as heart rate and breath rate). This shows that researchers today routinely select which (combinations of) signals are most promising for a given task.

## 2. Highlights

In the following paragraphs, we give an overview of the contributed manuscripts and their main contributions to the field. We structure them according to the cognitive processes which are in the respective focus of interest, according to the core themes identified in the previous section.

### 2.1. Cognitive workload

Gateau et al. perform a study in which they investigated cognitive workload (induced by a serial memorization task in two difficulty levels) in pilots from prefrontal fNIRS. They show that binary classification of workload level was feasible both in flight simulator as well as in a real aircraft. Additionally, the authors investigated the differences between these two conditions and revealed that workload tends to be higher in the real aircraft. Workload classification performances were similar in both conditions though. Grissmann et al. investigated in a study how the simultaneous stimulation of multiple cognitive and affective states, namely workload and valence, influenced the respective neural markers in the EEG signal. They found that while correlates of workload (in frontal theta and parietal alpha power) could still be detected, these correlates were affected by negative valence. Moreover they found no evidence of typical neural correlates of affect. This has implications for many real-world applications for which different concurrent mental states can be expected. In a study with 17 healthy volunteers, Aghajani et al. investigated mental workload classification using EEG and fNIRS. Workload was induced using a letter n-back task and could be classified using Support Vector machines in EEG, fNIRS, and the combination of both, which outperformed the individual modalities.

The impact of a multimodal approach for the classification of working memory load was also investigated by Liu et al.. These authors presented a study in which they discriminated three levels of mental workload, induced by an n-back task, using EEG, fNIRS, and physiological measures, with data combined over modalities performing best. They could show that all three modalities yielded better than chance level classification. To reduce calibration time for each subject, they demonstrated how results could be improved by learning from other subjects.

Extending offline analysis to a closed-loop system, Walter et al. demonstrate in their study that cognitive workload can be estimated from EEG to automatically adapt the difficulty level in a learning environment. The prediction model was trained on 10 subjects beforehand and no user-specific calibration data was needed, highlighting the feasibility in realistic scenarios. Labonte-Lemoyne et al. investigate a closed-loop system which adapted a game of Tetris to the cognitive load of the player, as measured from EEG and facial expression, with dynamic thresholding. In their user study, they show that adaptation to measured cognitive user states is not a trivial task and neither perceived experience, nor objective performance could be improved compared to the control condition.

### 2.2. Memory processes

Noh et al. used ERP features from the EEG signal to discriminate between successful and unsuccessful retrieval of memorized items. In their study, they show that the presence or absence of contextual information interacts with the type of information to be encoded. Tian et al. conducted an EEG study to investigate neural markers for the discrimination of low and high performers in a repeated memory task. They found that fronto-central spectral entropy during the retention period of working memory was predictive for intra-session and inter-session differences in performance. Toppi et al. use graph-theoretic connectivity features to discriminate different phases of working memory processing in 60-channel EEG data from a study in which 17 participants performed a Sternberg task. The authors could identify topological differences between the encoding, storage, and retrieval phases and also found a relationship between the neural markers and the participants' behavior.

### 2.3. Attention

Riccio et al. worked with a P3-BCI and study how attentional processing differs between patients suffering from amyotrophic lateral sclerosis (ALS) and healthy users and whether and how this corresponds to differences in P3-BCI performance. The corresponding study investigates attentional processing via a rapid serial visual presentation task. Results revealed that ALS patients have degraded attentional performances which translates in smaller P3 amplitude and lower P3-BCI classification performances. Using EEG, Baldwin et al. investigated mind wandering during simulated driving. In their study, they found increased alpha activity and reduced magnitude of P3a components to auditory stimuli in periods of mind wandering. As mind wandering during driving is a potential safety threat, this study can have large impact in future adaptive interfaces. Brouwer et al. presented a study combining EEG and eye tracking to investigate target encoding during visual search. They could show that eye tracking features distinguished better between hits and misses (i.e. targets which are never reported by the participant), while EEG features differentiated better between targets and non-targets. These results highlight how the two modalities complement each other.

### 2.4. Methods & other cognitive processes

Verdière et al. measure engagement of pilots via fNIRS in a flight simulator. In their study, different levels of engagement are induced via manual vs. automatic landing. The authors propose connectivity-based features and show that these can be used for effective classification. Takayoshi et al. used rewards in a simple number-discrimination task to study motivation and apathy tendency. In their study, they found P2 and P3 ERPs in EEG to be modulated by reward dependence and apathy tendency, which is an important step toward an adaptive interface based on motivation. Touryan et al. used a simultaneous visual search tasks and auditory n-back to investigate the neural sources of target detection in the presence of eye movements and during simultaneous task-demand. In their study, the authors used a modified measure-projection approach to separate the neural sources into six regions of interest (ROIs). This enabled efficient target detection from EEG signals, as well as revealing the time course of EEG activity in these ROIs—thus leading to a better understanding of the underlying processes. Nicolae et al. investigate a relatively novel concept by studying depth of processing (on three levels: no processing, shallow processing, or deep processing) from event-related time-domain and frequency domain features in EEG on a single-trial basis. In their study, they demonstrate the feasibility of this approach in three domains (namely: memory, language, and visual imagination).

## 3. Summary

The presented articles tackle a large variety of cognitive processes, their neurophysiological correlates and their detection. This research shows great progress in basic understanding of cognition as well as for applied research, especially for the development of adaptive systems. They also highlighted the need for more research into realistic and ecologically valid application environments of cognitive state estimation. If such states could be reliably estimated in real application scenarios–outside the lab—which is not really the case so far—then this could prove very useful for multiple applications such as aeronautics, driving, education or video games, among many others. Future directions of research should also include thorough attempts to define and differentiate the modeled concepts (e.g., how can we discriminate “mental workload” from “memory load”) and find a consensus on publicly available data sets for joint evaluations. A role model here could be the field of affective computing. Available common data sets such as DEAP (Koelstra et al., 2012), fNIRS-nback (Herff et al., 2014), EEG (So et al., 2017), or EEG combined with fNIRS (Shin et al., 2017) could be a start for such a program.

## Author contributions

FP coordinated the writing process and proposed the structure of the article. All authors contributed to the writing process.

### Conflict of interest statement

The authors declare that the research was conducted in the absence of any commercial or financial relationships that could be construed as a potential conflict of interest.
